# Biogeographical Ancestry Analyses Using the ForenSeq^TM^ DNA Signature Prep Kit and Multiple Prediction Tools

**DOI:** 10.3390/genes15040510

**Published:** 2024-04-18

**Authors:** Nina Mjølsnes Salvo, Gunn-Hege Olsen, Thomas Berg, Kirstin Janssen

**Affiliations:** Centre for Forensic Genetics, Department of Medical Biology, Faculty of Health Sciences, UiT The Arctic University of Norway, Post Box 6050, 9037 Tromsø, Norway

**Keywords:** genotyping performance, massively parallel sequencing, biogeographical ancestry, genetic prediction, human populations, forensic genetics

## Abstract

The inference of biogeographical ancestry (BGA) can assist in police investigations of serious crime cases and help to identify missing people and victims of mass disasters. In this study, we evaluated the typing performance of 56 ancestry-informative SNPs in 177 samples using the ForenSeq™ DNA Signature Prep Kit on the MiSeq FGx system. Furthermore, we compared the prediction accuracy of the tools Universal Analysis Software v1.2 (UAS), the FROG-kb, and GenoGeographer when inferring the ancestry of 503 Europeans, 22 non-Europeans, and 5 individuals with co-ancestry. The kit was highly sensitive with complete aiSNP profiles in samples with as low as 250pg input DNA. However, in line with others, we observed low read depth and occasional drop-out in some SNPs. Therefore, we suggest not using less than the recommended 1ng of input DNA. FROG-kb and GenoGeographer accurately predicted both Europeans (99.6% and 91.8% correct, respectively) and non-Europeans (95.4% and 90.9% correct, respectively). The UAS was highly accurate when predicting Europeans (96.0% correct) but performed poorer when predicting non-Europeans (40.9% correct). None of the tools were able to correctly predict individuals with co-ancestry. Our study demonstrates that the use of multiple prediction tools will increase the prediction accuracy of BGA inference in forensic casework.

## 1. Introduction

Biogeographical ancestry (BGA) is the geographical origin of a person’s ancestors based on population genetic structures [1]. BGA inference is a valuable intelligence tool in forensic genetics. Inferring a person’s BGA from crime scene DNA alone can aid police investigations by providing investigative leads and narrowing down a pool of potential suspects in cases where conventional DNA profiling fails to match a suspect or a DNA database record [2,3]. The intelligence tool can also aid in the identification of missing people and victims of mass disasters. 

BGA inference is possible by using ancestry informative markers (AIMs), which have alleles with different frequencies in various populations. In the forensic genetics context, AIMs are often short autosomal sequences, such as single nucleotide polymorphisms (SNPs), insertion/deletions (indels), and/or microhaplotypes [4,5,6]. Short markers are preferred because forensic samples often contain small amounts of DNA and/or degraded DNA. To be able to infer BGA beyond the continental level, many more markers are required than for conventional DNA profiling. High throughput methods such as massively parallel sequencing (MPS) have enabled the typing of a larger number of markers on very small amounts of DNA [7]. Over the last decade, various commercial and community-developed MPS assays that include AIM panels for BGA prediction have been made available, such as MAPlex [4], the Precision ID Ancestry Panel by Thermo Fisher Scientific, and the VISAGE Basic Tool [8,9]. Herein, we used the ForenSeq™ DNA Signature Prep Kit on the MiSeq FGx system (QIAGEN). This kit has been available for almost a decade now and has been proven to be a robust assay for obtaining reliable SNP profiles from low amounts of DNA [10,11]. Primer mix B, supplied in the kit, contains primers to multiplex over 200 forensically relevant genetic markers, including 56 ancestry informative SNPs (aiSNPs). The ForenSeq 56 aiSNP panel comprises the widely used Kidd lab (Yale University) panel of 55 SNPs [12] plus rs1919550. The 55 Kidd aiSNPs were selected based on their ability to predict ancestry on a continental scale. With relatively few SNPs, this panel is able to differentiate five to nine global biogeographic regions, depending on the number of reference populations used [12,13,14]. Integrated into the forensic MPS workflow with the ForenSeq kit, Universal Analysis Software (UAS) both analyses the sequencing data and performs BGA predictions. The predictions are made based on the 56 aiSNPs using a two-dimensional (2D) principal components analysis (PCA) plot with reference populations from the 1000 genomes phase 1 project.

As part of an in-house implementation of forensic BGA analysis, we evaluated the genotyping performance of the aiSNPs using the ForenSeq™ DNA Signature Prep Kit as well as the predictive performance of the UAS when typed in a Norwegian study population. The BGA prediction accuracy was furthermore compared to two other available prediction tools with overlapping SNP panels, the forensic research/reference on genetics knowledge base (FROG-kb) [15,16] and GenoGeographer [17]. FROG-kb calculates the relative likelihoods of ancestry on 160 reference populations, whereas GenoGeographer runs a likelihood ratio test on 36 reference populations. When inferring BGA for forensic casework, a multiple-tool approach is recommended [18,19]. In this study, we provide comparison and error assessments of three prediction tools that might be relevant for forensic laboratories. 

## 2. Materials and Methods

### 2.1. Study Population

Blood samples were collected from 730 volunteers (presumably unrelated) residing in northern Norway from 2015–2017. The ancestry of the volunteer’s grandparents was self-reported. The samples were divided into a reference set of 200 Norwegians (defined by four Norwegian grandparents) [14] and a test set of 530 individuals. Of the individuals in the test set, 503 had European ancestry (395 Norwegian), 22 had non-European ancestry (North African, Sub-Saharan African, South Asian, East Asian, Middle Eastern, and Siberian), and 5 had reported co-ancestry from either Europe and Asia or Europe and Africa. All samples were collected with fully informed consent and subsequently anonymized. The project was approved by the Faculty of Health Sciences, UiT, the Arctic University of Norway (reference number 2021/2034).

### 2.2. Library Preparation and Sequencing

All samples were previously genotyped with the ForenSeq™ DNA Signature Prep kit, Primer mix B (QIAGEN) on a MiSeq^®^ FGx instrument [20]. Batches of 32 libraries were loaded on each flow cell. To assess the technical sensitivity of the 56 aiSNPs, serial dilutions (500, 250, 125, 62.5, and 31.3 pg) of human male reference DNA 2800 M and 007 were analyzed in triplicates. Additionally, 2800 M was analyzed with 1000 pg and 007 with 15.6 pg input DNA in triplicates (see Salvo et al. [20] for more details). For the BGA analysis, only complete aiSNP profiles were analyzed. In cases with suspected drop-outs, the samples were retyped.

### 2.3. Analysis of the Sequence Data

Run metrics and sequence data were processed using the ForenSeq™ Universal Analysis Software v1.2 (UAS, QIAGEN), with interpretation criteria as described in Salvo et al. [20]. The default analytical threshold of 1.5% (minimum 10 reads) and interpretation threshold of 4.5% (minimum 30 reads) were applied for all loci. The assessment of the technical performance of the aiSNPs was performed using 177 samples from the same six representative sequencing runs as in Salvo et al. [20], with cluster densities of 1200–1600 K/mm^2^. The performance evaluation was based on profile completeness, read depth, and heterozygote balance. Heterozygote balances, also referred to as allele balances, were calculated for all heterozygous genotypes by dividing the number of reads for one allele with the number of total reads for the nucleotide position.

The description of the allele frequencies and Hardy-Weinberg equilibrium (HWE) analysis were performed using GenAlEx v6.5 [21]. 

### 2.4. Population Structure of the Norwegian Reference Population

Ancestral proportions of the Norwegian reference set (n = 200) were evaluated using STRUCTURE software version 2.3.4 [22,23,24,25] and a principal component analysis (PCA) using the ggbiplot package in R version 4.2.2. The structure of the Norwegian reference set was based on the 55 aiSNPs (Kidd panel) together with 31 reference populations (n = 2154) from West Africa (W Africa), Southwest Asia (SW Asia), Mediterranean Europe (Med. Europe), North Europe (N Europe), and West Siberia (W Siberia), kindly provided by Kenneth K. Kidd (Table S1). We applied the standard admixture model, assuming correlated allele frequencies. At each *K* value from 2 to 10, the program was run 20 times with 10,000 burn-ins and 10,000 Markov Chain Monte Carlo (MCMC) iterations. GenAlEx v6.5 [21,26] was used to prepare the input data file for STRUCTURE, Structure Harvester v.0.6.94 [27] was used to choose the most likely value of *K*, and Clumpak [28] was used to obtain averaged the Q-matrix data. The SNP rs1919550 was excluded in the STRUCTURE, and PCA analyses as the individuals in the reference populations were not typed for this marker.

### 2.5. Biogeographical Ancestry Prediction

Biogeographical ancestry (BGA) predictions for each individual in the test set (n = 530) were carried out using three different prediction tools: UAS, FROG-kb, and GenoGeographer. Prior to the ancestry analysis, the Norwegian reference set (n = 200) was included as a reference population in FROG-kb and GenoGeographer. This was not possible for the UAS.

### 2.6. UAS

The initial BGA inference was performed using the UAS with the default parameters. The UAS obtains ancestry estimations of unknown samples through PCA using 1000 genome data based on the ForenSeq 56 aiSNPs. The results from several 1000 genome populations were clustered into 3 major ancestry groups (African, East Asian, and European). An Ad Mixed American cluster was also included in the PCA plot as a reference. The BGA estimation of the unknown sample was possible if it clustered with one of the major ancestry clusters. If the unknown sample was plotted outside any of these clusters or clustered within the Ad Mixed American cluster, we considered the estimation as inconclusive (see the example in Figure S1).

### 2.7. FROG-kb

The standalone Java application, FrogAncestryCalc [16], was applied to directly access the underlying FROG-kb data (http://frog.med.yale.edu/FrogKB/ accessed on 2 February 2024) [15] and to run an ancestry likelihood function to assemble the matrix of likelihoods on the 55 Kidd aiSNPs of 160 potential ancestral populations (underlying reference populations). When compared to the unknown sample, the underlying reference populations were ranked based on the likelihood of origin. The higher-ranked populations were presented as the more likely population of origin. However, population likelihoods within one order of magnitude were not considered significantly different [15]. Hence, the population ranked as the most likely population of origin was not necessarily the correct one, and lower-ranked populations could not be excluded. Considering this, all populations within one order of magnitude were considered for the predictions. In the case of ambiguous results (e.g., if the populations within one order of magnitude originated from different continents), the prediction was considered inconclusive.

### 2.8. GenoGeographer

GenoGeographer Version 0.1.14 (http://apps.math.aau.dk/aims/ accessed on 19 August 2021) [17] was applied using the meta-populations, a 95% confidence interval (CI), and Kidd loci (55 Kidd aiSNPs). The reference populations were grouped into nine metapopulations plus the Norwegian reference set. GenoGeographer performed a statistical z-score test, a likelihood ratio test, for each aiSNP profile from the test set to assess whether the profile likely originated from any of the reference populations in the database [17]. When using a one-sided 95% CI, the critical z-score value was 1.64. Z-scores ≤ 1.64 indicate that the unknown profile is likely to originate from the reference population, and it is “accepted” as the most likely population of origin. On the contrary, a sample is “rejected” if it does not obtain z-scores ≤ 1.64 for any of the populations in the reference set. The unknown individual can have a z-score ≤ 1.64 for more than one population. If two or more populations are accepted, a likelihood ratio (LR) is computed, which can be used together with the CI to evaluate which population is significantly more plausible than the others. If the accepted populations were not significantly different, the predictions were considered inconclusive (similar to Mogensen et al. [29]).

## 3. Results

### 3.1. Genotyping Performance of the aiSNPs Using the ForenSeq™ DNA Signature Prep Kit

**Figure 1:**
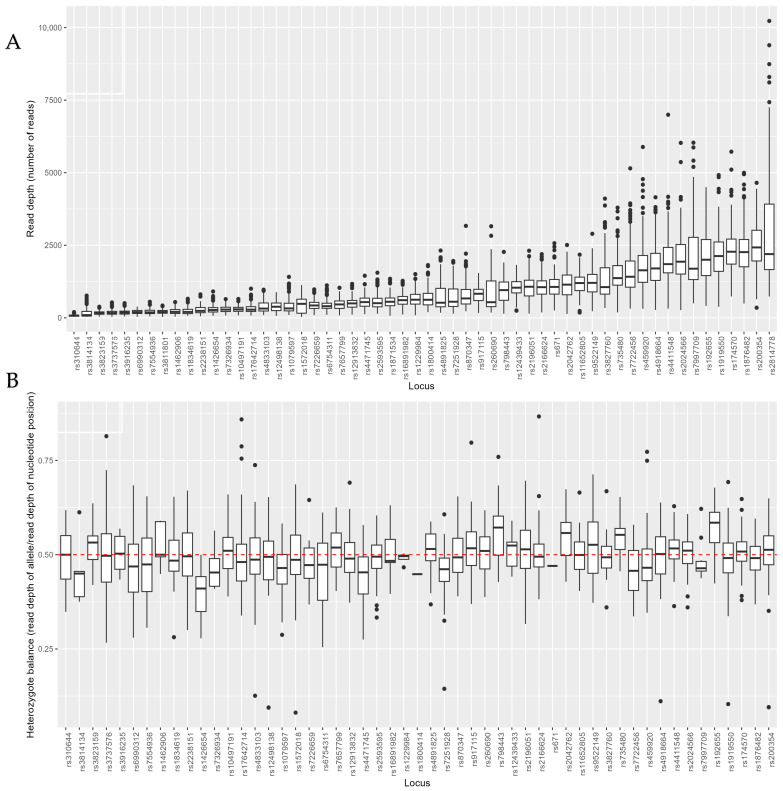
(**A**) Read depth and (**B**) heterozygote balance (read depth of allele/read depth of nucleotide position) of the 56 aiSNPs genotyped with the ForenSeq™ DNA Signature Prep Kit (n = 177).

The technical performance of the ForenSeq multiplex was evaluated based on all 56 aiSNPs in 177 samples from six different sequencing runs, of which all had a cluster density higher than 1200. In total, 9876/9912 (99.64%) individual locus genotype calls were made. Out of the 177 samples typed, 146 (82.49%) generated complete aiSNP profiles. Drop-outs/reads below the analytical threshold were observed in three SNPs, rs3814134, rs310644, and rs1572018, which failed to type in 19 out of 177 (10.73%), 16 out of 177 (9.04%), and 1 out of 177 samples (0.56%), respectively (Table S2). The markers rs310644 and rs3814134 had median read depth across samples of less than 100 reads (73 and 94 reads, respectively, Figure 1A and Table S2). In total, 23 of 56 (41%) markers showed low read depth with minimum reads <100 (Figure 1A, Table S2). The heterozygote balance varied from 0.13 to 0.74 in the SNP with the largest range (rs4833103, range = 0.67, n = 78), and from 0.47 to 0.50 in the SNP with the smallest range (rs1229984, range = 0.04, n = 4, Figure 1B and Table S3). Three of the aiSNPs were homozygous in this data set (rs1871534, rs2814778, and rs3811801), and heterozygote balance calculations were not applicable for these (Table S3). Figure 1 shows that the median of the heterozygote balances in most loci was close to the expected 0.5. There was no clear pattern indicating that loci with higher read depths have less variable heterozygote balances.

**Figure 2:**
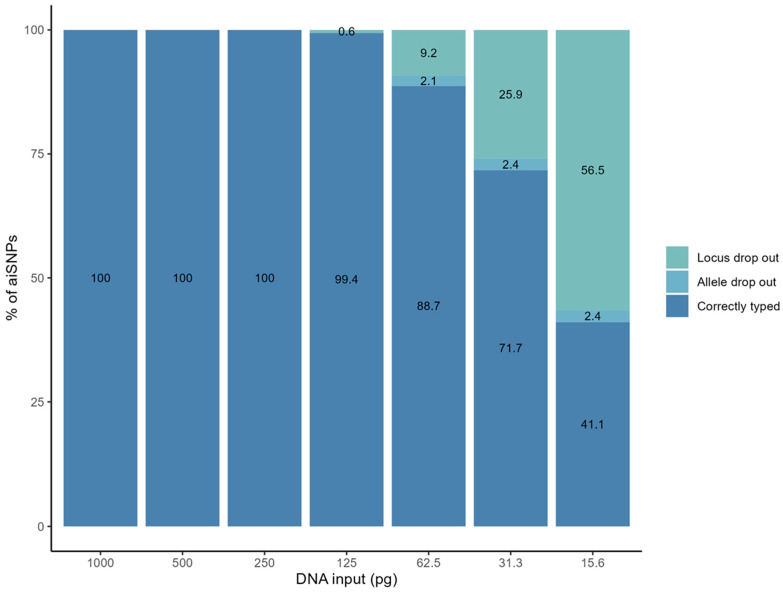
Sensitivity study of the 56 aiSNPs genotyped with the ForenSeq™ DNA Signature Prep Kit, showing the profile completeness in relation to the DNA input.

To assess the kit`s ability to produce full profiles from low DNA input amounts, a dilution series of control DNA was sequenced. The read depth per locus decreased nearly linearly with decreasing amounts of input DNA, and heterozygote balances became more variable with decreasing amounts of input DNA (Figure S2). For the control DNA, the success rate over all runs was 100% down to 250 pg and 99.4% at 125 pg (Figure 2). At 125 pg, loci rs3737576 and rs310644 dropped out in two samples. At 62.5 pg, 15 loci and seven alleles dropped out. The allele drop-out was observed in rs12913832, rs10497191, rs2238151, and rs1572018. The SNP rs1572018 and rs310644 had the overall highest number of drop-outs across all the dilutions, in 13 of 36 (36%) and 12 of 36 (33%) samples, respectively.

False homozygous aiSNP profiles were observed in 19 SNP genotypes in low-input samples with less than 250 pg DNA. In total, 17 alleles were below the default analytical threshold of 10 reads and were, therefore, not called. The detected alleles in the seemingly homozygous profiles were above the interpretation threshold of 30 reads, ranging from 31 to 89 reads (average = 50 reads). The remaining two allele drop-outs were due to poor balance (<0.1). 

### 3.2. Genetic Structure of the Norwegian Reference Population

Prior to the prediction analysis using FROG-kb and GenoGeographer, a Norwegian reference population was added to the underlying reference data (see Table S4 for the allele frequencies and calculations of the HWE). This population has previously been included in a study on the genetic relationship of European, Mediterranean, and Southwest Asian populations, analyzed with global reference populations using the 55 aiSNP Kidd panel [14]. For a more “zoomed in” perspective, we herein present the genetic structure of the Norwegian reference population when analyzed with mainly European populations (Table S1). 

**Figure 3:**
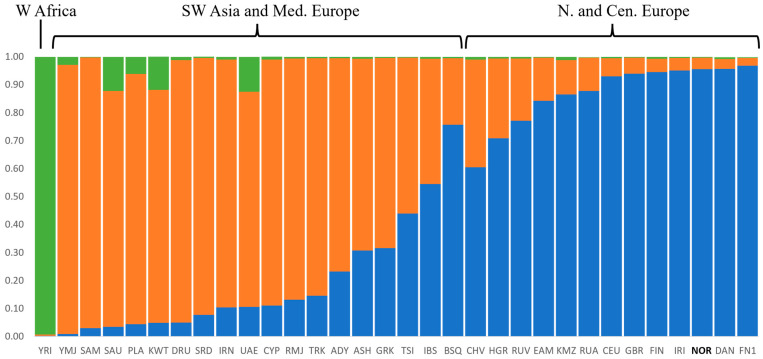
STRUCTURE analysis with *K* = 3 using 55 aiSNPs and 32 reference populations (Table S1). “Admixture” and ”correlated allele frequency” models were considered in the analysis.

To assess the genetic admixture patterns of the Norwegian population, STRUCTURE and PCA analyses were performed using 32 populations (n = 2354) from North-Central Europe (including the Norwegian reference population, NOR, and one population from West Siberia, KMZ), Mediterranean and Southwest Asia, and one outlier population from West Africa (Table S1). Based on the delta *K* calculations [30], *K* = 3 was the optimal number of *K* for the STRUCTURE analysis (Figure S3). The estimated cluster membership values as average population Q-values for the highest likelihood results are shown in the stacked bar plot in Figure 3. Clustering was observed among West African (green bar), Southwest Asian (orange bar), and Northern European populations (blue bar). Populations in Central and Mediterranean Europe showed both orange and blue clusters. The blue cluster also encompasses the population from West Siberia (the Komi Zyriane, KMZ). The Norwegian population had averages (Q-values) of 95.6% European, 4.1% Southwest Asian, and 0.3% West African origin, and could not be differentiated from other Northern European populations. 

**Figure 4:**
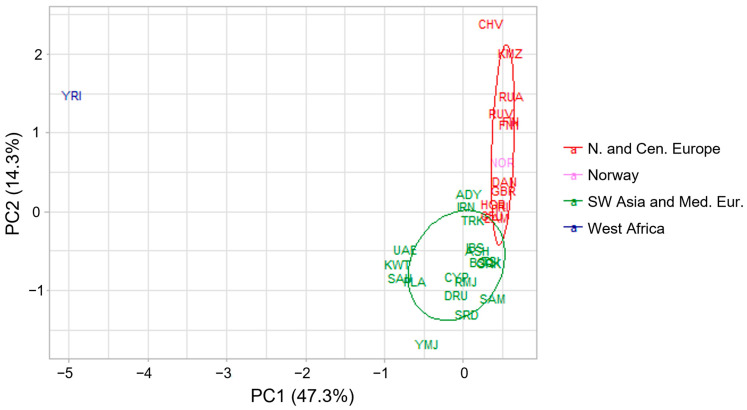
PCA results based on the 55 aiSNP (Kidd panel) allele frequencies for 32 reference populations (including the Norwegian reference population), Table S1.

The PCA plots showed similar results as the STRUCTURE analysis (Figure 4). The first principal component (PC1) separated the West African population from the rest. The second PC showed a clinal organization of the Southwest Asian to the Mediterranean to the North European populations. The Norwegian population overlapped with the North European populations, which infers that the Norwegian population is an admixture of North European populations. Thus, if a Norwegian individual is predicted based on the 55 Kidd aiSNPs to be of Northern European origin, it should be considered correct. 

### 3.3. Biogeographical Ancestry Prediction

**Figure 5:**
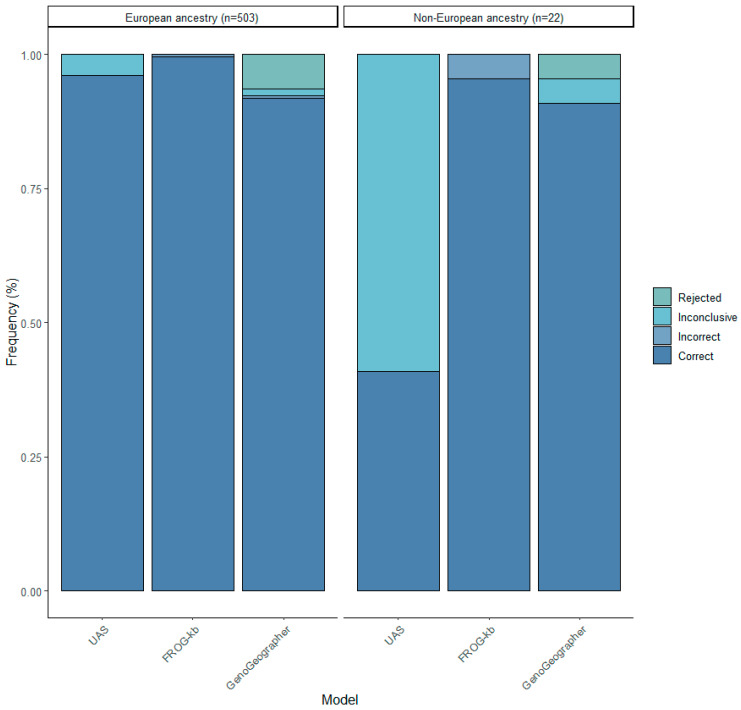
BGA predictions obtained from the three prediction tools, UAS, FROG-kb, and GenoGeographer, using 503 individuals with European ancestry and 22 individuals of non-European ancestry.

Three different ancestry prediction tools were assessed based on whether an individual was assigned to the correct population or not. The test population consisted of 503 Europeans (395 Norwegians), 22 non-Europeans, and 5 individuals with co-ancestry from more than one continent. Figure 5 shows the prediction accuracies of the three tools predicting the individuals of European and non-European ancestry.

The individual aiSNP profiles were initially analyzed with the UAS using the default parameters to predict the most likely ancestry. In total, 96.0% (483/503) of the European samples were correctly predicted, whereas only 40.9% (9/22) of the non-Europeans were correct (Figure 5). Of the non-Europeans, the model could only predict individuals with sub-Saharan African and East Asian ancestry. The remaining samples (20 European and 13 non-European) were considered inconclusive as they clustered with the Ad Mixed American cluster or outside any clusters. 

FROG-kb had the highest rates of correct predictions, in 99.6% (501/503) of the Europeans and 95.4% (21/22) of the non-Europeans (Figure 5). However, although low, the model also had the highest number of incorrect predictions, in three samples. Out of the incorrect predictions, two were of European (one Russian and one Norwegian) and one was of North African ancestry, all predicted to be Asians. No sample was considered inconclusive. 

For the individual ancestry assignment by GenoGeographer, 91.8% (462/503) of the Europeans and 90.9% (20/22) of the non-Europeans were “accepted” to the correct population (Figure 5). Two European individuals were incorrectly predicted. One of these was a Norwegian who was predicted to be North African and was not the same Norwegian who was incorrectly predicted with FROG-kb. The other was incorrectly predicted to be East Asian and was the same European that was incorrectly predicted using FROG-kb. In total, seven Europeans and one Asian were considered inconclusive because they were accepted to more than one meta-population with an overlapping CI. GenoGeographer had the overall lowest correct rate with thirty-two Europeans and one non-European being “rejected”. When only considering the samples that were not rejected by the model, in total, 98.1% (462/471) of the Europeans and 95.2% (20/21) of the non-Europeans were correctly predicted. Notably, 23 of the 32 rejected European samples were correctly predicted by both the UAS and FROG-kb.

None of the prediction tools were able to correctly predict the five individuals with reported co-ancestry from two continents. Using the UAS, three individuals were inconclusive and two were incorrectly predicted to be European or African. Using FROG-kb, all five individuals were incorrectly predicted to be Asian, European, or African. The GenoGeographer tool rejected one individual with reported co-ancestry from Europe and Africa, and incorrectly predicted the remaining four individuals to be either Middle Eastern, West Greenlander, or admixed European/Middle Eastern/South-Central Asian. 

## 4. Discussion

In this study, we evaluated the technical performance of the 56 aiSNPs in the ForenSeq multiplex and demonstrated that it is highly sensitive. Confirming previous reports, it produced full BGA profiles with input DNA down to 250 pg [10,11]. However, when evaluating the technical performance of the 177 samples from six high-performing sequencing runs, we observed low read depths and occasional drop-outs in loci rs3814134, rs310644, and rs1572018 in samples with the recommended DNA input (1 ng). Another locus with low read depth, rs3737576, was the first to drop-out in the low-input samples. The low performance (amplification efficiency) of these SNPs is not unique to our study [31,32,33,34]. Frégeau et al. [33] detected a correlation between the amplicon length, AT/GC content, and read depth. Short amplicons with AT-rich content, such as rs310644, might have lower PCR efficiency and, thereby, lower read depth. In a previous study, we suggested to increase the interpretation threshold from 30 to 100 reads for phenotype-informative SNPs (piSNPs) in this kit when analyzing low-input samples [20]. As several aiSNPs in our population dataset had low read depths (below 100 reads) even with a 1ng input, increasing the interpretation threshold would lead to a high loss of aiSNP genotypes, e.g., rs310644 would drop-out in 76% (123/161) of the samples in the present study. Care should be taken when predicting ancestry on partial profiles as drop-outs might cause misleading predictions. To avoid drop-outs, we therefore suggest striving to use the recommended DNA input (1 ng) for BGA analysis. In this study, all samples with suspected drop-outs were retyped to obtain complete aiSNP profiles for the BGA analysis. 

By comparing the different forensic BGA tools’ ability to assign an individual to its correct population, we show that the UAS, FROG-kb, and GenoGeographer tools performed similarly well when predicting individuals of European ancestry. When predicting individuals of non-European ancestry, FROG-kb and GenoGeographer performed better than the UAS. A prediction could only be made using the UAS if the unknown sample clustered within one of the three major ancestry groups (African, East Asian, and European) in the PCA plot, posing a challenge in predicting other non-European samples, e.g., Middle Eastern and Asian samples. Consequently, 59% of the non-Europeans in our study population could not be predicted using the UAS. When utilizing the FROG-kb, we were able to predict all individuals except one of the non-Europeans and two of the Europeans, who were inconclusive when using the UAS. The FROG-kb has more underlying reference populations than the UAS, and instead of using a PCA, it calculates the relative likelihoods for each reference population and ranks them from highest to lowest. This makes the results easier to interpret, and no inconclusive predictions were produced. However, a relative likelihood model will assign the unknown sample to the least unlikely population, regardless of whether the correct population is present in the reference data or not, which might lead to misleading predictions. In our study, the FROG-kb produced three incorrect predictions. Despite the high number of global reference populations, the FROG-kb reference data is not exhaustive, which could contribute to some errors. To overcome the challenge of non-exhaustive reference data, GenoGeographer employs a likelihood ratio test, similar to an outlier test [35]. Using the likelihood ratio test, the model will “reject” the unknown sample if it is not “similar enough” to any of the reference populations. Herein, the GenoGeographer performed similarly to FROG-kb with two incorrect predictions. It is important to note that inaccuracy can also be due to limitations in the SNP panel used or errors in the self-reported population affiliation. However, the stringent criteria to be “accepted” led to 33 “rejected” samples (32 Europeans) using GenoGeographer, and thus a lower correct rate than the FROG-kb. As we were only using metapopulations to predict BGA, the high reject rate might be because of non-sufficient homogeneity in the metapopulations, causing the underlying populations to be rejected even if they are correct. 

Five of the individuals in our sample population had reported co-ancestry from two different continents (Europe and Asia or Europe and Africa). Of these, only two had first order of admixture (1:1 admixture ratio of the first generation of admixed parents). As expected, the UAS, FROG-kb, and GenoGeographer tools were not able to predict these samples correctly as the tools are not designed to handle co-ancestry with this SNP panel. Notably, it is possible to choose to analyze the first order of admixture using GenoGeographer. However, our two individuals with first order of co-ancestry were not predicted correctly as they were both predicted to be Middle Eastern. To assess co-ancestry using autosomal markers, STRUCTURE is well-recognised in the field as a reliable tool [36]. However, due to long simulation runs, the analysis is time-consuming and might not be the first choice for BGA analyses in routine casework. If utilized, it is advised to use a comprehensive reference dataset and to carefully consider the parameters set in STRUCTURE, as these can influence the BGA inference [19]. Moreover, supplementation with uniparental DNA analysis (Y and mtDNA) would elevate the possibility of accurately detecting ancestral admixtures [3].

Based on the observations in this study and in line with other studies, we concur to combine several prediction tools when conducting BGA analyses [18,19]. The UAS is a user-friendly software that integrates the analysis of sequence data with immediate BGA predictions. Because the software is integrated into the MPS workflow, it is highly suitable for initial BGA inferences in forensic casework, especially for European populations. However, because of its limitations in predicting non-Europeans, additional tools should complement the UAS. Based on our study sample, we demonstrated that the FROG-kb and GenoGeographer tools accurately predict BGA using the 55 Kidd aiSNPs genotyped with the ForenSeq™ DNA Signature Prep Kit. Additionally, these two tools are user-friendly and freely available. However, the reference data and statistics used by the prediction tool can evidently influence the prediction outcome and should be thoroughly evaluated by any forensic laboratory before implementation.

## 5. Conclusions

In this study, we demonstrated that the ForenSeq™ DNA Signature Prep Kit produces highly reliable aiSNP profiles using the MiSeq FGx system. However, the user should be aware that some SNPs showed, on average, lower read depth than others, which can lead to drop-outs. For forensic BGA analyses, it is advised to analyze complete aiSNP profiles. Therefore, we suggest not using less than 1 ng of input DNA (the recommended DNA input for the ForenSeq kit) for BGA analyses. Additionally, we demonstrated that the FROG-kb and GenoGeographer tools are highly reliable BGA tools for the prediction of European and non-European individuals using the 55 Kidd aiSNP panel. The UAS was highly reliable when predicting individuals of European ancestry. However, because of the limited underlying reference data, the UAS could not infer over half of the non-Europeans typed in this study. Therefore, we highly recommend supplementing the initial BGA analysis using UAS with FROG-kb and/or GenoGeographer. Notably, none of these tools could correctly predict individuals with co-ancestry, which could be possible by performing additional analyses such as STRUCTURE.

## Data Availability

The original contributions presented in the study are included in the article and Supplementary Materials, further inquiries can be directed to the corresponding authors.

## Supplementary Materials

The following supporting information can be downloaded at: https://www.mdpi.com/article/10.3390/genes15040510/s1, Table S1: Reference populations for STRUCTURE and PCA analysis; Table S2: Descriptive statistics in number of reads of the 56 aiSNPs genotyped with the ForenSeq™ DNA Signature Prep Kit; Table S3: Descriptive statistics for heterozygote balance calculations of the 56aiSNPs typed using ForenSeq™ DNA Signature Prep Kit; Table S4: Allele and genotype frequencies and summary of Chi-Square Tests for Hardy-Weinberg Equilibrium of the 55 aiSNPs in the Norwegian reference population; Figure S1: PCA plot obtained from the UAS, demonstrating predictions considered inconclusive; Figure S2: Sensitivity study of the 56 aiSNPs genotyped with the ForenSeq™ DNA Signature Prep Kit. (A) Read depth and (B) heterozygote balance; Figure S3: Delta K calculations [30] plotted using Structure Harvester on STRUCTURE results of the Norwegian reference population (n = 200) together with 31 other populations (n = 2154) (Table S1). References [13,30,37] are cited in the Supplementary Materials.
