# Supplementary material for: Biogeographical Ancestry Analyses Using the ForenSeqTM DNA Signature Prep Kit and Multiple Prediction Tools
Source: Genes (Basel). 2024 Apr 18;15(4):510. doi: 10.3390/genes15040510 (PMC11050699; doi:10.3390/genes15040510)
Supplement: Supplementary file 1 [file genes-15-00510-s001.zip › Supplement figures for ancestry manuscript.pdf]

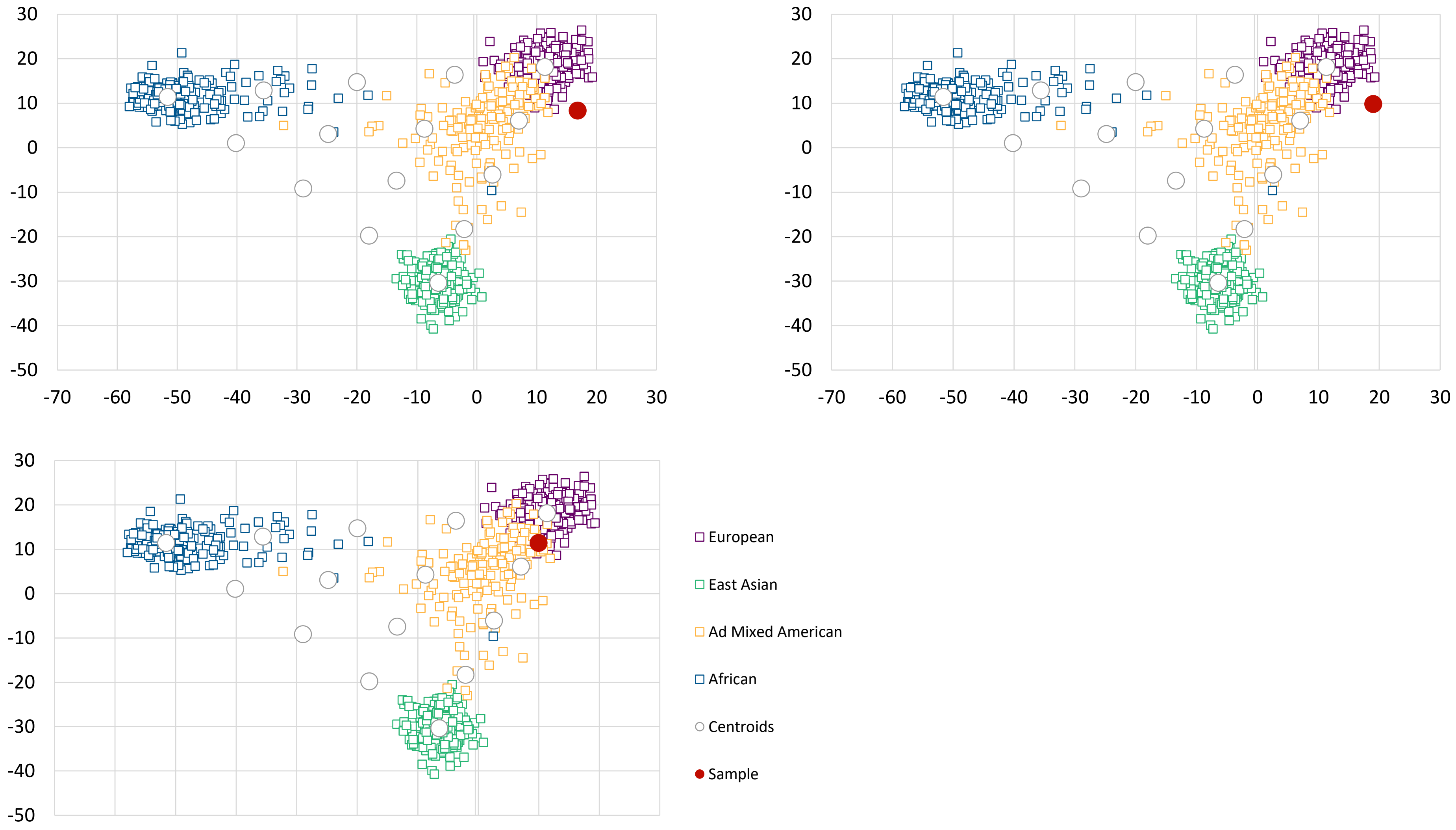

Figure S1: PCA plot obtained from the UAS, demonstrating predictions considered inconclusive

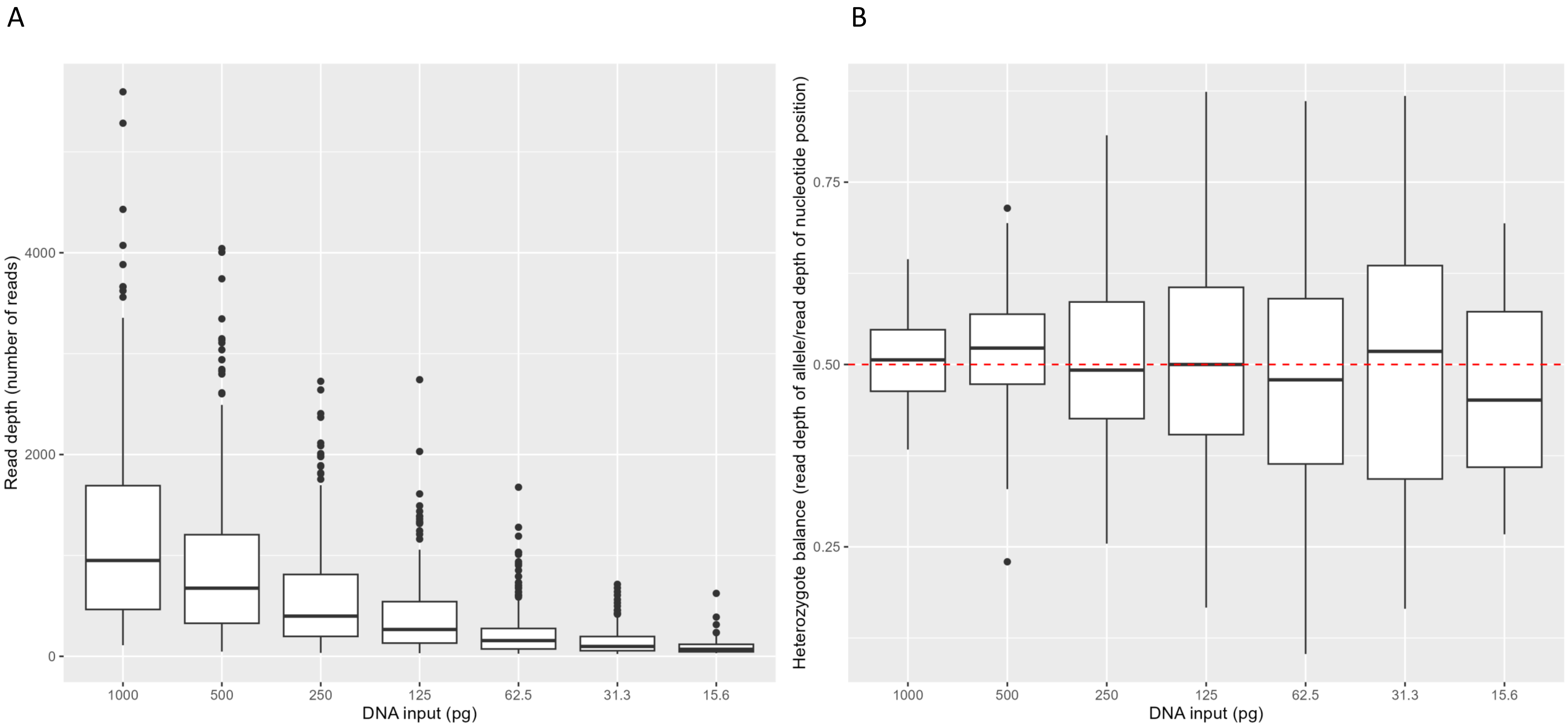

Figure S2: Sensitivity study of the 56 aiSNPs genotyped with the ForenSeq™ DNA Signature Prep Kit.
A) Read depth and B) heterozygote balance.

A

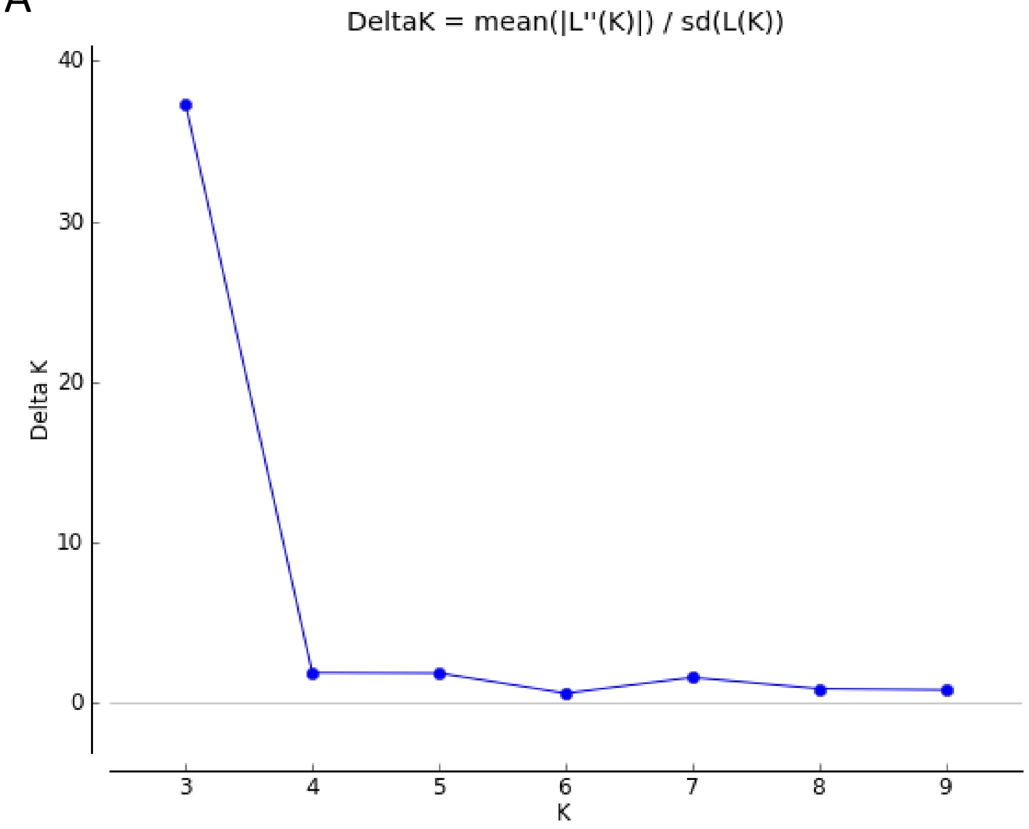

Figure S3: Delta *K* calculations (EVANNO et al., 2005) plotted using Structure Harvester on STRUCTURE results of the Norwegian reference population (n=200) together with 31 other populations (n=2154) (Table S1)
